# When can total knee arthroplasty be safely performed following prior arthroscopy?

**DOI:** 10.1186/s12891-020-03859-1

**Published:** 2021-01-04

**Authors:** Jin-Ning Ma, Xiao-Lin Li, Pan Liang, Sheng-Li Yu

**Affiliations:** Department of Orthopaedic Surgery, Ningxia People’s Armed Police Corps Hospital, No. 895, Qinghe South Street, Xingqing District, Yinchuan, 750001 Ningxia province China

**Keywords:** Total knee arthroplasty, Arthroscopy, Clinical outcome, Survivorship

## Abstract

**Background:**

The optimal timing to perform a total knee arthroplasty (TKA) after knee arthroscopy (KA) was controversial in the literature. We aimed to 1) explore the effect of prior KA on the subsequent TKA; 2) identify who were not suitable for TKA in patients with prior KA, and 3) determine the timing of TKA following prior KA.

**Methods:**

We retrospectively reviewed 87 TKAs with prior KA and 174 controls using propensity score matching in our institution. The minimum follow-up was 2 years. Postoperative clinical outcomes were compared between groups. Kaplan-Meier curves were created with reoperation as an endpoint. Multivariate Cox proportional hazards regressions were performed to identify risk factors of severe complications in the KA group. The two-piecewise linear regression analysis was performed to examine the optimal timing of TKA following prior KA.

**Results:**

The all-cause reoperation, revision, and complication rates of the KA group were significantly higher than those of the control group (*p* < 0.05). The survivorship of the KA group and control group was 92.0 and 99.4% at the 2-year follow-up (*p* = 0.002), respectively. Male (Hazards ratio [HR] = 3.2) and prior KA for anterior cruciate ligament (ACL) injury (HR = 4.4) were associated with postoperative complications in the KA group. There was a non-linear relationship between time from prior KA to TKA and postoperative complications with the turning point at 9.4 months.

**Conclusion:**

Prior KA is associated with worse outcomes following subsequent TKA, especially male patients and those with prior KA for ACL injury. There is an increased risk of postoperative complications when TKA is performed within nine months of KA. Surgeons should keep these findings in mind when treating patients who are scheduled to undergo TKA with prior KA.

## Introduction

Although the American Academy of Orthopaedic Surgeons guidelines suggest there is no benefit in the knee arthroscopy (KA) for knee osteoarthritis (KOA) [1], KA is still frequently performed in these patients to improve clinical symptoms and delay total knee arthroplasty (TKA) intervention [2]. About 2 million KAs were performed each year globally for KOA, and this number is dramatically increasing over time [2–4]. A recent systematic review has indicated an annual rate of progression to TKA of 2.6% [5]. Thus, it is inevitable to perform a TKA in a patient with a prior KA for contemporary arthroplasty surgeons.

Several studies have indicated the prior KA had a deleterious effect on the subsequent TKA, including higher incidences of complication, revision, and periprosthetic joint infection (PJI) [6–8]. Therefore, With the goal of improving outcomes following TKA in patients with prior KA, it’s critical to identify who may be not suitable for a TKA. Moreover, whether previous KA has a time-dependent effect on subsequent TKA remains an inconclusive but important question [7–9].

Therefore, the purpose of this study was to 1) explore the effect of prior KA on subsequent TKA; 2) identify who were not suitable for TKA in patients with prior KA, and 3) determine the timing of TKA following prior KA.

## Methods

After Institutional Review Board approval, we retrospectively reviewed 92 primary TKAs with the clinical history of prior KA from January 2013 to 2017 in our institution. We excluded patients with a history of septic arthritis and those with other procedures on the ipsilateral knee. The minimum follow-up was 2 years. After the aforementioned exclusion criteria, 87 TKAs were enrolled as the KA group. Each patient in the KA group was matched to two controls without the prior surgical procedure of any kind using propensity score matching (PSM). The detail of matching was in the statistical analysis.

Patient demographic characteristics, including age at the timing of TKA, gender, BMI, and American Society of Anesthesiologists (ASA) score and the year from KA to TKA were reviewed. All patients had cemented posterior-stabilized (PS) Vanguard TKA (Zimmer Biomet, Warsaw, Indiana). The outcomes included Hospital for Special Surgery (HSS) score, range of motion (ROM), stiffness, venous thromboembolism (VTE), periprosthetic fracture, all-cause reoperation, all-cause revision, and periprosthetic joint infection (PJI). The Diagnosis of PJI was according to the Musculoskeletal Infection Society criteria for infection [10]. The postoperative stiffness, VTE, and PJI were considered severe complications.

### Statistical analysis

The KA subjects were matched with controls at a 1:2 ratio using PSM according to the nearest neighbor matching without replacement within a caliper width of 0.1. Parameters were chosen for inclusion in the PSM calculation, including gender, age, BMI, ASA score, diagnosis, and year of surgery. The balance of covariates between groups was examined by calculating standardized mean differences (SMD).

Date on patients’ demographics and outcomes were compared between groups with the Mann-Whitney test for continuous variables and the chi-square test for categorical variables. Kaplan-Meier curves were created with reoperation as time-to-event outcomes. The differences in survivorship between groups were compared using the log-rank test. Multivariate Cox proportional hazards regressions were performed to identify risk factors of severe complications in the KA group. Adjusted smoothing spline plots were created to graphically depict thetime-dependent effect of KA on severe complications following subsequent TKA. Then the two-piecewise linear regression analysis was performed to examine whether there was a threshold effect or not. All statistical analyses were performed with the statistical software packages R (http://www.R-project.org, The R Foundation).

## Result

Patient characteristics were shown in Table 1. The PSM yielded 87 TKAs in the KA group and 174 TKAs in the control group. The age of KA and the control group was (63.1 ± 7.9, 47–73) year and (63.0 ± 7.6, 51–75). The quality of PSM was considered balanced (all SMD < 0.1). The preoperative ROM was 108 ± 18.3 for the prior KA group and 111 ± 19.6 for the control group (*p* = 0.127), and the hip-knee-ankle (HKA) angle was 9.5 ± 6.3 for the prior KA group and 8.1 ± 4.6 for the control group (*p* = 0.081). The reasons for KA prior to TKA were for KOA (41, 47.1%), meniscus tears (21, 24.1%), chondromalacia (13, 14.9%), and anterior cruciate ligament (ACL) injury (12, 13.8%).

**Table 1:** Patient characteristics

Demographics	KA group (*n* = 87)	Control group (*n* = 174)	SMD	*P*
Age (mean ± SD)	63.1 ± 7.9	63.0 ± 7.6	0.0012	0.983
Male (*n*, %)	37 (42.5%)	75 (43.1%)	0.0063	0.930
BMI (mean ± SD)	27.9 ± 4.6	27.7 ± 4.9	0.0039	0.912
ASA (mean ± SD)	2.3 ± 0.9	2.3 ± 0.7	0.0015	0.946
Diagnosis			0.0029	0.883
Osteoarthritis	72 (82.8%)	146 (83.9%)	–	–
Rheumatoid arthritis	9 (10.3%)	17 (9.8%)	–	–
Posttraumatic arthritis	6 (6.9%)	11 (6.3%)	–	–

**Fig. 1:**
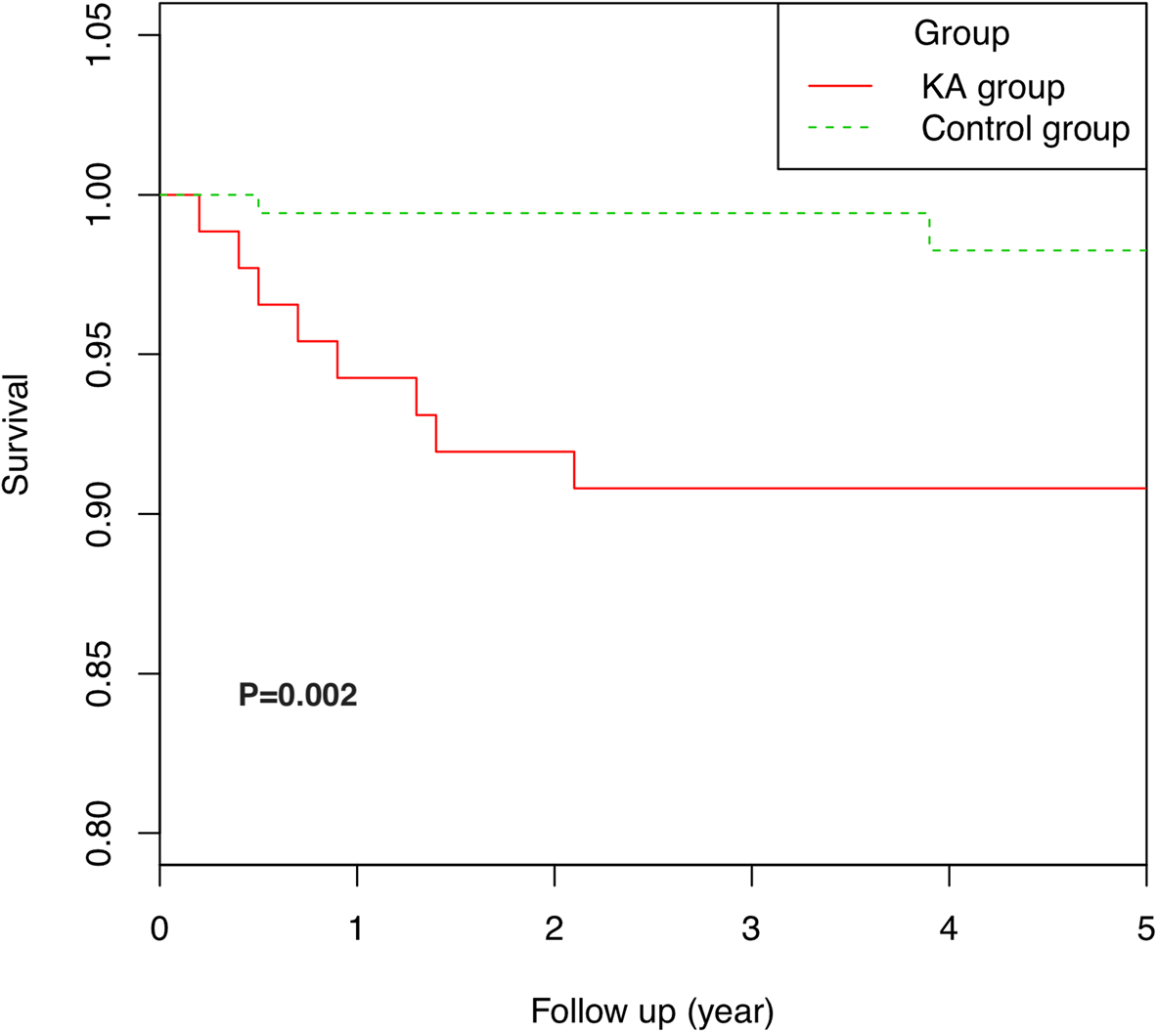
Survivor curve for the KA group and control group with reoperation as an endpoint

The mean follow-up was 4.3 ± 1.9 years. The outcomes of the two groups were present in Table 2. The all-cause reoperation and revision rates of the KA group were significantly higher than those of the control group. There was no difference in HSS score ROM and VTE between groups. Patients in the KA cohort had higher incidences of stiffness and PJI. With all-cause reoperation as an endpoint, the survivorship for KA group and control group was 92.0% (95% CI, 86.4–97.9%) and 99.4% (95% CI, 98.3–100%) at the 2-year follow-up (*p* = 0.002), respectively (Fig. 1). There were five revision TKAs in the KA group, including 42-stage exchange arthroplasties for PJI and one aseptic revision for the knee’s instability. Three cases had wound healing problems and underwent debridement in the KA group. One case in the control group had periprosthetic fracture with loosening femoral component and underwent revision at one year. Another patient in the control group had the knee’s stiffness at 5 months and underwent arthroscopic release.

**Table 2:** Outcomes between KA group and control group

	KA group	Control group	*P*
HSS score	94.1 ± 8.7	94.8 ± 9.1	0.075
ROM	110 ± 15.7	112 ± 13.1	0.069
All-cause reoperation	8 (9.2%)	2 (1.1%)	**0.004**
All-cause revision	5 (3.4%)	1 (0.6%)	**0.028**
Severe complication	11 (12.6%)	4 (2.3%)	**0.002**
Stiffness	5 (5.7%)	1 (0.6%)	**0.028**
VTE	2	3	0.873
PJI	4 (4.6%)	0	**< 0.001**

**Fig. 2:**
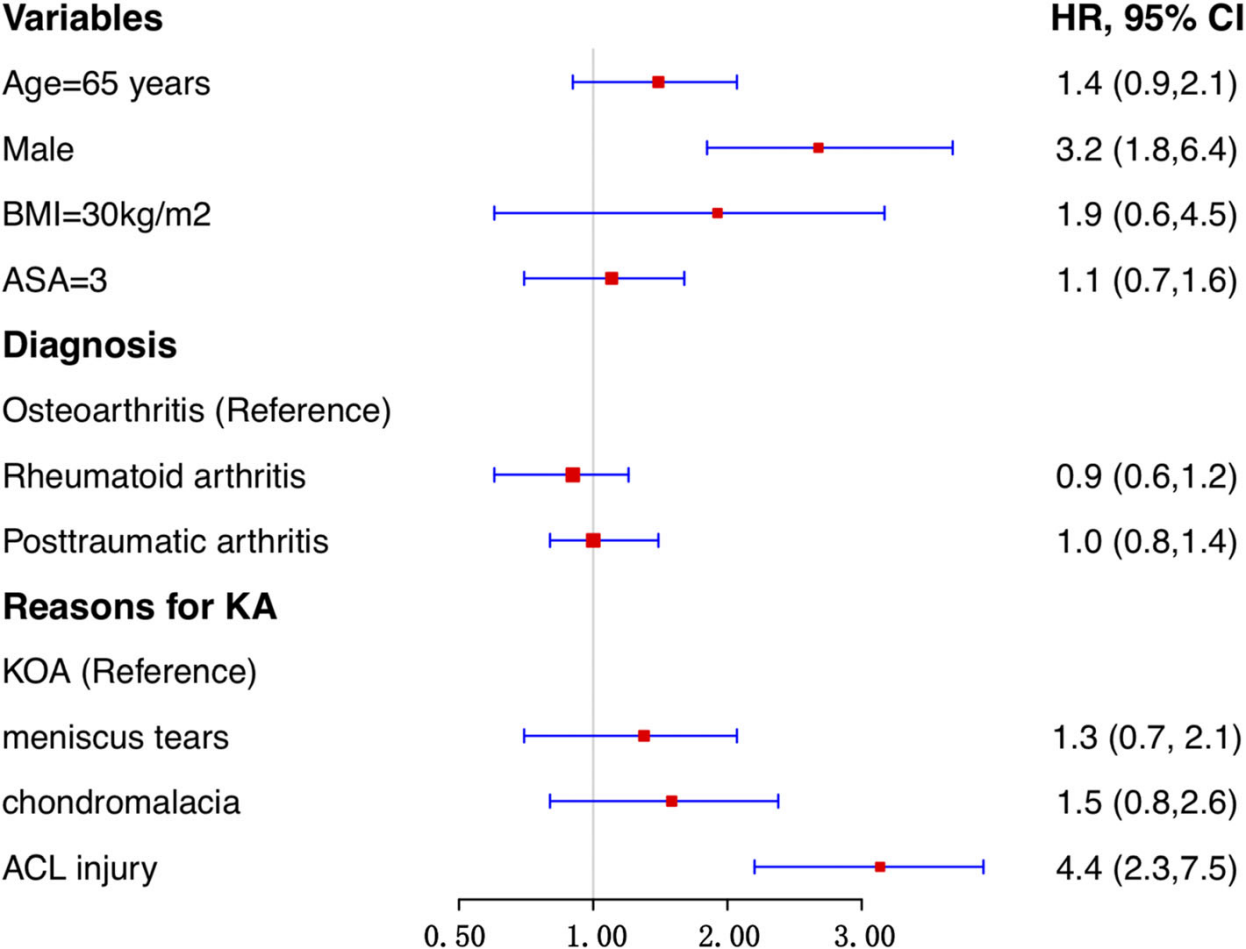
Risk factors for severer complications following TKA in KA the group

The risk factors of reoperation in the KA group were shown in Fig. 2. After adjusting potential confounders, male patients and patients with prior KA for ACL injury had a higher risk of reoperation following subsequent TKA. Other variables were not associated with reoperation.

**Fig. 3:**
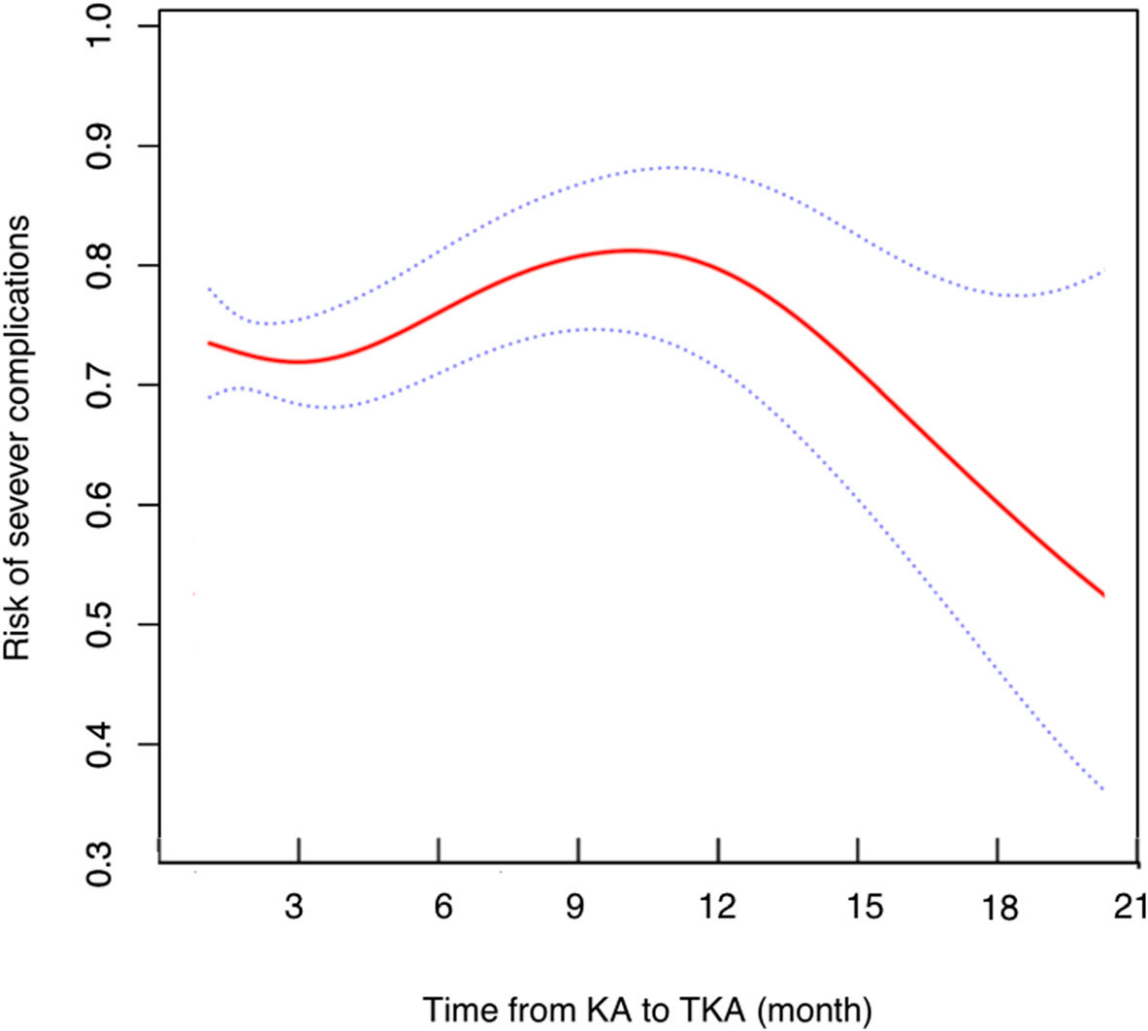
The smoothing spline plots present the non-linear relationship between time to TKA and severe complications

The average time from KA to TKA was (2.3 ± 1.7) years. The adjusted smoothing spline (Fig. 3) suggested a non-linear relationship between the time from KA to TKA and severe complications. The two-piecewise linear regression analysis indicated the risk of severe complications decreased with time to TKA more than the turning point at 9.4 months.

## Discussion

In this PSM based control study, patients with a prior KA had a higher risk of reoperation, revision, stiffness, and PJI than controls following subsequent TKA. Male and prior KA for ACL injury were independent risk factors of severe postoperative complications in patients with prior KA. The time-dependent effect analysis suggested it was more reliable to perform a TKA at least 9 months after the prior KA.

Our result presented inferior clinical outcomes in patients with prior KA, which was consistent with the most recent study. Alex et al. utilized the Humana insurance database to review 3357 TKAs with a prior KA and 134,662 controls. The multivariate analysis suggested the prior KA was associated with a higher prevalence of revision, postoperative stiffness, and PJI [6]. However, Viste et al. reported conflicting results [4]. They retrospective reviewed a single institutional database, including 160 TKAs, had a prior KA. They matched a control cohort at a 1:2 ratio and compared Knee Society Score (KSS), ROM, complications, and survivorships with a mean follow-up of 9 years. They found the clinical outcomes of TKAs with prior KA were comparable with that of controls.

To be our best knowledge, although several studies have evaluated the effect of prior KA on TKA, there was no study to identify risk factors of worse outcomes in these patients. The present study found males and KA for ACL injury were associated with postoperative complications in patients with prior KA. Male patients had worse outcomes, as males may be more active than females. Several studies have suggested TKA after ACL reconstruction resulted in worse outcomes following TKA. Watters et al. reviewed 122 patients with prior ACL reconstruction with a minimum of 2-year follow-up. They indicated TKA with a prior ACL reconstruction had a higher risk of longer operative time and early reoperation [11]. Chong et al. performed a retrospective study, including 101 cases with prior ACL reconstruction and 202 controls [12]. However, they found there was no statistical difference in estimated blood loss and postoperative complications between the ACL group and controls. The potential reason for the higher risk of PJI and stiffness in TKA patients with a prior KA may be due to the history of multiple surgeries.

It’s critical to explore the time-dependent effect of prior KA on the subsequent TKA to determine the timing of TKA. The optimal time to perform a TKA after KA was controversial in the literature. A study by Piedade et al. reviewed 60 primary TKA with a prior KA and 1119 controls, and no time-dependent effect was found [9]. However, both Werner et al. and Barton et al. recently reported patients who underwent TKA within six months after KA had worse Patient-Reported Outcome and a higher risk of postoperative complications [7, 8]. The most potential limitation of the two studies was to determine the cutoff of time arbitrarily. Considering time from KA to TKA as a continuous variable, the present study created smoothing spline plots and conducted the two-piecewise linear regression analysis to explore the timing of TKA. We found patients who were scheduled to undergo TKA should wait at least 9 months after KA.

There are several limitations to the present study. First, the study design was retrospective in nature and thus was subject to its inherent biases, such as a recall bias. Second, although we tried our best to identify patients with prior KA through medical records and institutional databases, we may miss several cases. Third, the sample size may be inadequate, and the possibility of a type-II error exists. Fourth, we did not analyze the Patient-Reported Outcome as only the HSS score was available before 2018 in my institution.

## Conclusions

Prior KA is associated with worse outcomes following subsequent TKA, especially male patients and those with prior KA for ACL injury. There is an increased risk of postoperative complications when TKA is performed within nine months of KA. Surgeons should keep these findings in mind when treating patients who are scheduled to undergo TKA with prior KA.

## Data Availability

The datasets used and/or analyzed during the current study are available from the corresponding author on reasonable request.
